# Effectiveness of an educational intervention to improve antibiotic dispensing practices for acute respiratory illness among drug sellers in pharmacies, a pilot study in Bangladesh

**DOI:** 10.1186/s12913-018-3486-y

**Published:** 2018-08-31

**Authors:** Fahmida Chowdhury, Katharine Sturm-Ramirez, Abdullah Al Mamun, A. Danielle Iuliano, Mohammod Jobayer Chisti, Makhdum Ahmed, Mejbah Uddin Bhuiyan, Kamal Hossain, Mohammad Sabbir Haider, Shaikh Abdul Aziz, Mahmudur Rahman, Eduardo Azziz-Baumgartner

**Affiliations:** 1 International Centre for Diarrhoeal Disease Research, (icddr,b), Bangladesh, Dhaka, Bangladesh; 2 Centers for Disease Control and Prevention (CDC), Atlanta, USA; 3 Institute of Epidemiology, Disease Control and Research, (IEDCR), Dhaka, Bangladesh

**Keywords:** Drug sellers, Pharmacy, Acute respiratory illness, Antibiotics, Dispensing practices, Educational intervention

## Abstract

**Background:**

Inappropriate dispensing of antibiotics for acute respiratory illness (ARI) is common among drug sellers in Bangladesh. In this study, we evaluated the impact of an educational intervention to promote guidelines for better ARI management among drug sellers.

**Methods:**

From June 2012 to December 2013, we conducted baseline and post-intervention surveys on dispensing practices in 100 pharmacies within Dhaka city. In these surveys, drug sellers participated in 6 standardized role-playing scenarios led by study staffs acting as caregivers of ARI patients and drug sellers were blinded to these surveys. After the baseline survey, we developed ARI guidelines and facilitated a one-day educational intervention about ARI management for drug sellers. Our guidelines only recommended antibiotics for children with complicated ARI. Finally, we conducted the six month post-intervention survey using the same scenarios to record changes in drug dispensing practices.

**Results:**

Only 2/3 of participating pharmacies were licensed and few (11%) of drug sellers had pharmacy training. All the drug sellers were male, had a median age of 34 years (IQR 28–41). For children, dispensing of antibiotics for uncomplicated ARI decreased (30% baseline vs. 21% post-intervention; *p* = 0.04), but drug sellers were equally likely to dispense antibiotics for complicated ARI (15% baseline vs. 17% post-intervention; *p* = 0.6) and referrals to physicians for complicated ARIs decreased (70% baseline vs. 58% post-intervention; *p* = 0.03). For adults, antibiotic dispensing remained similar for uncomplicated ARI (48% baseline vs. 40% post-intervention; *p* = 0.1) but increased among those with complicated ARI (44% baseline vs. 78% post-intervention; *p* < 0.001). Although our evidence-based guidelines recommended against prescribing antihistamines for children, drug sellers continued to sell similar amounts for uncomplicated ARI (33% baseline vs. 32% post-intervention; *p* = 0.9).

**Conclusions:**

Despite the intervention, drug sellers continued to frequently dispense antibiotics for ARI, except for children with uncomplicated ARI. Pairing educational interventions among drug sellers with raising awareness about proper antibiotic use among general population should be further explored. In addition, annual licensing and an reaccreditation system with comprehensive monitoring should be enforced, using penalties for non-compliant pharmacies as possible incentives for appropriate dispensing practices.

**Electronic supplementary material:**

The online version of this article (10.1186/s12913-018-3486-y) contains supplementary material, which is available to authorized users.

## Background

In low and middle-income countries, drug sellers in pharmacies play an important role as healthcare providers by offering health advice, treatment recommendations and antibiotics for common ailments [1, 2]. Bangladesh has a severe shortage of qualified healthcare providers [3, 4].People with limited resources often resort to seeking healthcare for general health problems from drug sellers at pharmacies [3, 5]. In the capital city, Dhaka, two out of five persons seek healthcare treatment from community pharmacies [6]. Community pharmacies are also the preferred point of care for acute respiratory illness (ARI) [7–10]. Although most ARIs are self-limited and caused by respiratory viruses [11], drug sellers often recommend and dispense antibiotics without prescription [12–18]. Even though the Bangladesh’s 2005 National Drug Policy prohibits drug sellers from dispensing antibiotics without a physician’s prescription, this law is not enforced [19]. In Bangladesh, drug sellers are usually not trained to obtain clinical histories or provide medical advice, and often fail to refer patients with severe ARI to a licensed medical provider for evaluation and treatment [10, 17, 20, 21]. A recent study among drug sellers in Dhaka noted that inappropriate dispensing and poor referral practices were common for patients with ARI [22, 23]. Although it is favourable to identify and refer patients with severe ARI to licensed physicians early on so that they may receive effective and appropriate treatment [24, 25], this does not often occur [23]. Moreover inappropriate dispensing of antibiotics by drug sellers can exacerbate antimicrobial resistance [26, 27]. Antimicrobial resistance has outpaced the development of new antimicrobial agents and is an important public health threat [28].

In Bangladesh, approximately 50% of pharmacies are unlicensed (not government approved) [29]. Pharmacy licenses are provided to drug sellers by the Directorate General of Drug Administration (DGDA), Government of Bangladesh, when they have completed a grade C pharmacy degree (i.e. three-month course) which grants them permission to legally dispense drugs [19]. A three-month self-learning pharmacy course, designed and jointly conducted by the Bangladesh Pharmaceutical Society and Bangladesh Chemists and Druggists Society, is available for $30 and eligible for persons having completed 10th grade and above. Those who pass the final examination receive a certificate of ‘C’ grade pharmacists. According to the Bangladeshi Drugs Act, no pharmacy can be given a license or allowed to dispense drugs without having a registered trained pharmacist (‘C’ grade pharmacist). Nevertheless, the DGDA rarely closes unlicensed pharmacies because it does not have adequate resources to identify non-compliant pharmacies [29].

Proper ARI management includes history taking, clinical examination when appropriate, illness counselling, treatment with appropriate medication, and timely referral [20, 30]. Educational interventions designed to improve drug-dispensing, referral practices and the reduction of inappropriate medication use have been tested among drug sellers in other countries [20, 26, 31–35]. Some studies have shown that these educational interventions can be effective [20, 32], while others have not [35].

In this study, we evaluated the impact of ARI management training on a previously surveyed population of drug sellers in Dhaka, Bangladesh [23].

## Methods

### Study design and setting

**Fig. 1:**
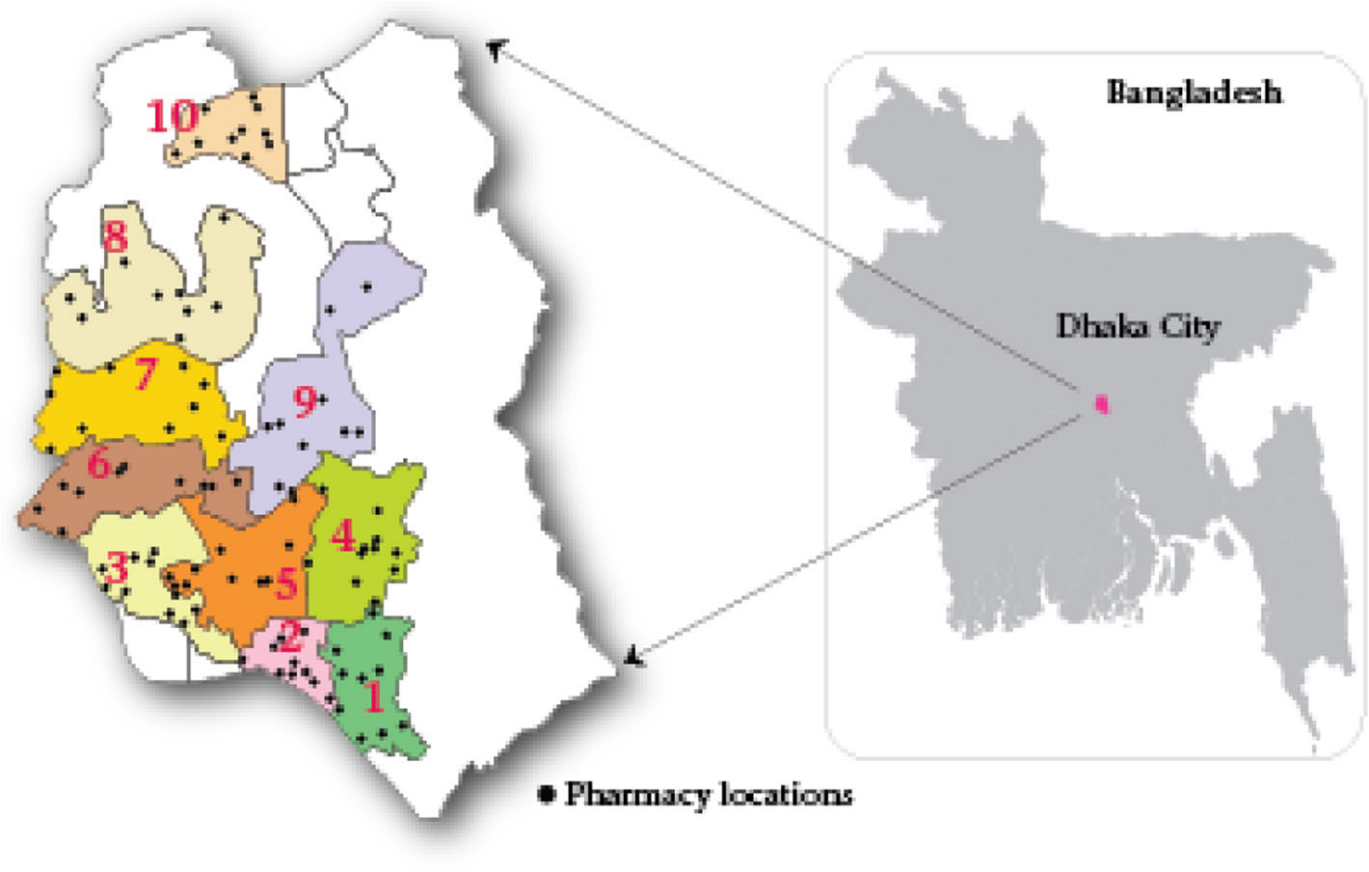
Pharmacy locations

To evaluate the effectiveness of the educational intervention, we conducted a baseline survey, and developed and disseminated a one-day ARI management training for 100 randomly selected pharmacies in Dhaka city from June 2012 to December 2013. Six months after the training, we conducted the post-intervention survey. Dhaka city has an estimated population of 8.5 million with approximately 9000 pharmacies [36] within 300 km^2^ [37]. Two study staff selected 10 pharmacies from each of the 10 zones in Dhaka city through randomly generated GPS coordinates as previously described [23] (Fig. 1).

### Pharmacy selection criteria

Study staff identified the closest pharmacy to each of the 100 randomly selected GPS coordinates. They selected any licensed (government approved) or unlicensed pharmacy which had been in operation for > 5 years to increase the likelihood that the pharmacy had a solid customer base. To better focus the assessment on dispensing practices of the drug seller without a physician’s prescription, the pharmacy also had to be > 100 m from a healthcare facility. Once the pharmacy was identified, the team enrolled the drug seller who worked the most hours in the pharmacy and had at least 2 years experience in dispensing medicine to customers with or without a prescription from a physician, to ensure the most exhaustive and comprehensive information.

### Data collection

After obtaining written informed consent, study staff interviewed the drug sellers using structured questionnaires to collect information about the drug sellers’ demographics. As part of this study, other study staff role-played as caregivers of an ARI patient (proxy consultation) and conducted a baseline and post-intervention assessment to describe drug sellers’ advice and drug dispensing practice for the management of ARI. Drug sellers were not informed of this assessment so to capture actual dispensing practices.

### Baseline survey

**Fig. 2:**
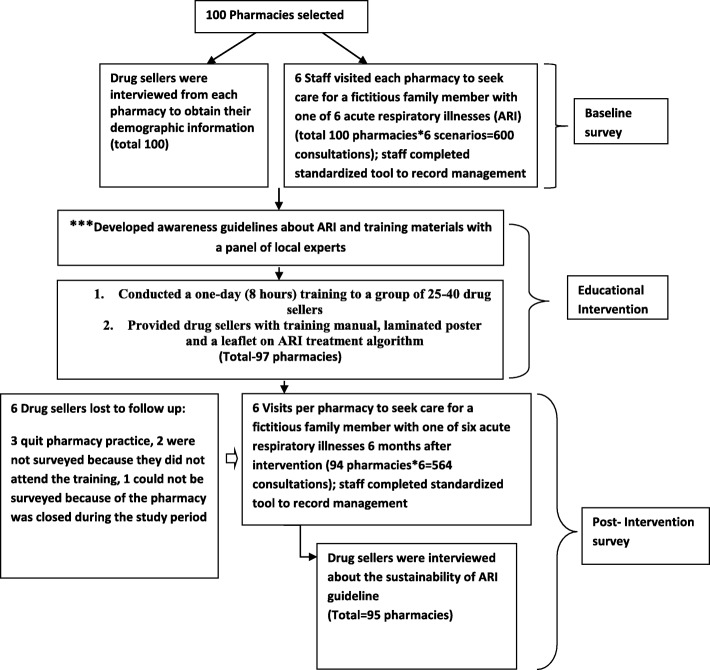
Flowchart on study steps forward

Six study staff role-played as caregivers for patients with ARI at the selected pharmacies. Before visiting pharmacies, the study staff received role-play training on how to request advice for the treatment of a family member from one of three adult scenarios or one of three child scenarios (Table 1). Each study staff was assigned a specific ARI scenario to role play at pharmacies. Study staffs conducted each pharmacy visits six times with the six predefined ARI scenarios within 14 days (Fig. 2).

**Table 1:** Features described by study staffs for ARI scenarios by age group

Adults	Children^a^
Cough and runny nose	Cough and runny nose
Fever with cough	Fever with cough
Fever with cough and breathing difficulty	Breathing difficulty

^a^If asked by the drug sellers, field staff stated the child’s age was 6 months, 1.5 years, 3 years, or 5 years in chronological order for each subsequent visit

### Defining uncomplicated and complicated ARI

Although staff role-played six scenarios, these were analyzed as either uncomplicated or complicated ARI. The first two scenarios of cough, runny nose and/or fever were considered uncomplicated ARI as these would only merit symptomatic treatment. The scenarios with breathing difficulty were considered complicated ARI whose treatment might benefit from antibiotics or should prompt a referral to a physician [24, 38]. If the drug sellers asked about the duration of specific symptoms for any of the scenarios, caregivers reported three days of fever, seven days of cough, and/or one day of respiratory distress when applicable. To make the scenario seem more realistic and to explore whether the drug seller sold medicines before their expiry date, study staff purchased them at the end of the consultation and did not present the same scenario to the same drug seller twice. Using a structured questionnaire, staff recorded information about the drug sellers’ history-taking, health advice, and dispensed drugs.

### Development of ARI guideline for drug sellers

After conducting the baseline survey and analyzing the data, we developed guidelines for drug sellers on the management of ARI, in consultation with stakeholders and local subject matter experts. Consultants included a panel of pediatric and adult respiratory medicine specialists, respiratory illness research scientists from International Centre for Diarrhoeal Disease Research, Bangladesh (icddr,b), the WHO project manager for the Integrated Management of Childhood Illness (IMCI) and the Director of the Institute of Epidemiology, Disease Control & Research (IEDCR) of the Government of Bangladesh. The guidelines provided information on the classification of ARI as complicated or uncomplicated among children and adults, and what drug sellers should offer customers: (1) self-care advice, (2) self-care advice and medication, or (3) referral to a physician or hospital (Table 2) (Additional file 1) and (Additional file 2). The guidelines for children (0–59 months) were based on existing World Health Organization IMCI recommended management of ARI [24]. As there are no existing guidelines for adult ARI management, our developed guidelines have recommended the symptomatic treatment of adults with over the counter (OTC) drugs and the referral of complicated ARI patients to physicians.

**Table 2:** Treatment recommendations for acute respiratory illness (ARI) according to our developed guidelines

Clinical scenarios for ARI	Treatment Recommendation
Uncomplicated ARI in children (< 5 years)	▪ No antibiotics ▪ Only symptomatic treatment -- If febrile, treatment with acetaminophen, sponging of body, hydration for patients ≥6 months -- For cough, drink warm lime water, honey, lemon tea -- Follow-up in 5 days if symptoms have not improved
Complicated ARI in children (< 5 years)	▪ Antibiotic for 5 days ▪ Follow-up in 2 days ▪ Advise the caretaker to return immediately if the child develops any danger signs (inability to drink or breastfeed, repeated vomiting, convulsion, lethargy/abnormally sleepy/unconscious) ▪ Refer urgently to physician/hospital if child develops danger sign
Uncomplicated ARI in adults	▪ No antibiotic ▪ Only symptomatic treatment -- Over the counter drugs such as cough syrups and antihistamines can be given; acetaminophen should be given if febrile -- For cough, drink warm lime water, honey, lemon tea or salt water gargling -- Follow-up in 5 days if symptoms have not improved
Complicated ARI in adults	▪ No antibiotics ▪ Referral to a physician

### Training the drug sellers / intervention

After developing these guidelines for drug sellers, we conducted a one-day group training at icddr,b in the local language (Bengali) for all the drug sellers from the 100 selected pharmacies. We conducted six group trainings with approximately 25–40 drug sellers in each group. We also provided drug sellers a laminated poster with the ARI management algorithm to hang in their pharmacies, laminated cards with the algorithm and training manuals about the guidelines to remind them of the recommended practices (Fig. 2).

### Post-intervention survey

Six months after the training, six different study staff visited the participating pharmacies with the same scenarios presented during the baseline survey and acted as caregivers for ARI patients to assess post-intervention ARI management practices (Fig. 2).

### Post-intervention survey on knowledge, attitude and practice of the drug sellers

On a subsequent and final visit, two study staff interviewed the drug sellers to ask about the feasibility of long-term compliance with the guidelines and to observe the status of the poster provided during our training (Fig. 2).

### Statistical analysis

For socio-demographic characteristics of the drug sellers, we described the data using frequency and percentage for all the categorical variables. We presented median and inter-quartile ranges (IQR) for the continuous variables when distributions were asymmetric. We compared the frequency with which drug sellers dispensed drugs (e.g. antibiotics and over-the-counter medications for symptom relief), made physician referrals and provided certain pieces of advice at baseline versus post-intervention using linear regression models adjusting for the dependency of the drug sellers’ responses.

### Ethics consideration

The study protocol was reviewed and approved by the institutional review boards (IRB) of icddr,b. CDC relied on icddr,b’s IRB review and provided assistance and guidance with the technical aspects of this study.

## Results

### Characteristics of the participants

Of the 100 participating pharmacies, 67 were licensed by the Government of Bangladesh, and 33 were operating without a license. Fifty-eight pharmacies had one single drug seller working at any given time. All the drug sellers were male, had a median age of 34 years (IQR 28–41), 12 years of education (IQR 10–14) equivalent to the completion of secondary school, 12 years of work experience (IQR 7–14) and generally worked 13 h a day (IQR 12–14). Less than half (48%) of the drug sellers had taken at least one accredited professional health care-related certification course, and 8% had taken more than one course. The median duration of accredited training was 6 months (IQR 6–12). Less than a third (28%) of drug sellers attended the “Local Medical Assistant and Family Planning” (LMAF) courses offered by the government or private institutions intended to train community members about the management of common illnesses such as ARIs and diarrhoea. Thirteen percent attended other training courses including “Rural Medical Practitioner” or “Paramedic” which are six-month to one-year courses and “Medical Diploma” which is a three-year course; none of these courses including LMAF are accredited by the Bangladesh Medical and Dental Council or grant students clinical privileges to treat patients as qualified healthcare providers. Only 11% of study participants attended the government accredited formal “pharmacy” course which is a requirement by the Government of Bangladesh to operate a pharmacy.

Of the drug sellers in the 100 participating pharmacies, only three did not attend the training for the management of ARI. One drug seller quit the pharmacy practice between the baseline and post-intervention survey and two could not attend the training (Fig. 2).

Azithromycin (12%; 138/1164 consultations) was the most commonly sold antibiotic followed by amoxicillin (11%; 129/1164). However apart from antibiotics, antihistamines (33%;383/1164) were frequently sold among the symptomatic drugs.

### Post intervention changes in antibiotic dispensing

#### Children with uncomplicated ARI

**Fig. 3:**
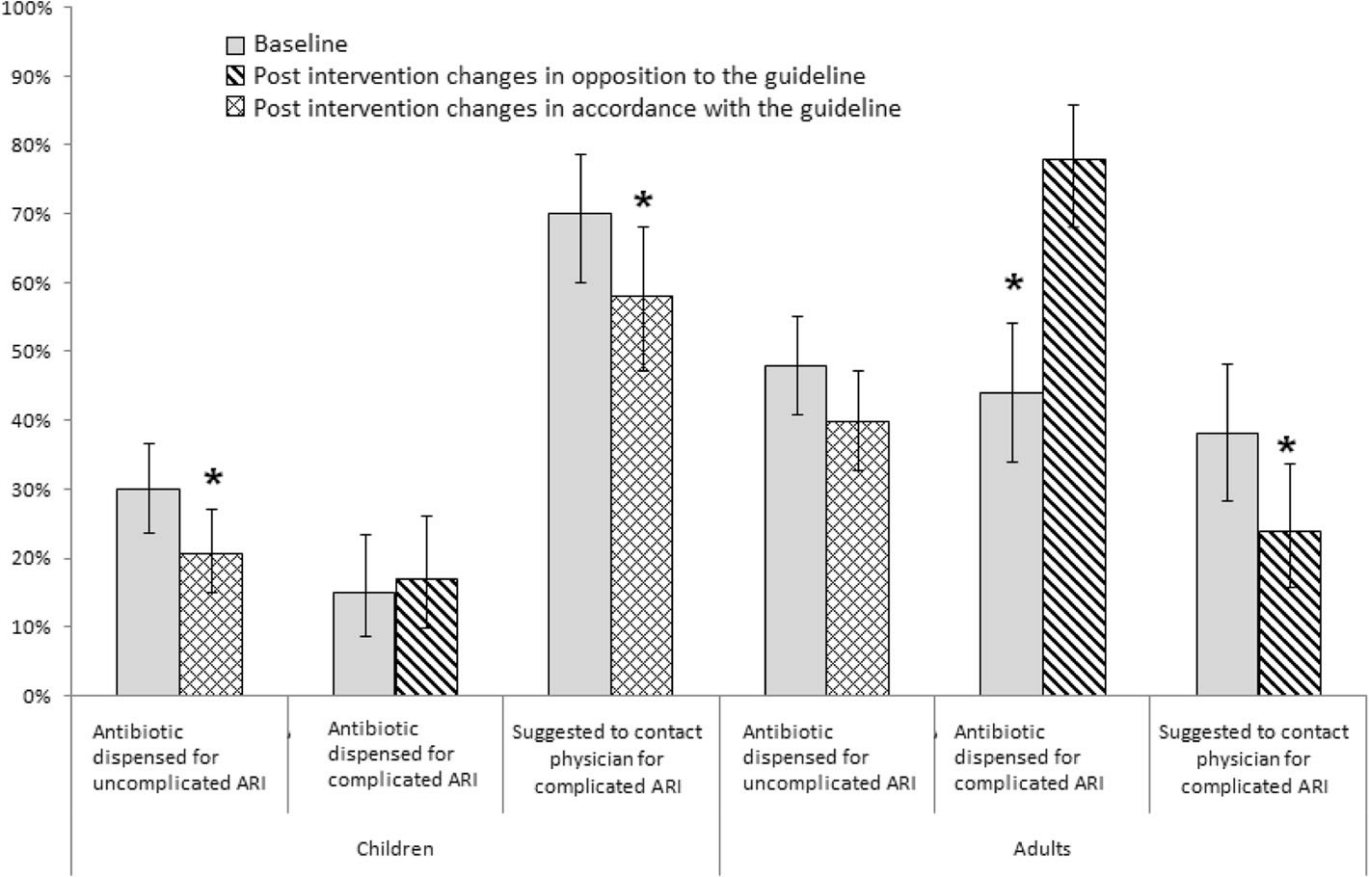
Antibiotic dispensed and patient referrals for acute respiratory illnesses(ARI) at baseline and post-intervention at selected Dhaka city pharmacies, June 2012 to December 2013

After the intervention, which recommended against the sale of antibiotics for uncomplicated ARI, antibiotic dispensing decreased for proxy consultations (30% [60/200 consultations] at baseline vs. 21% [39/188 consultations] post-intervention; *p* = 0.04) (Fig. 3).

#### Children with complicated ARI

Although the guidelines recommended selling antibiotics for the treatment of complicated ARI, the proportion of consultations where drug sellers sold antibiotics remained unchanged (15% [15/100 consultations] at baseline vs. 17% [16/100consultations] post-intervention; (*p* = 0.6)] (Fig. 3). The proportion of consultations where drug sellers followed intervention recommendations to refer children with severe pneumonia for medical evaluation decreased (70% at baseline vs. 58% post-intervention; *p* = 0.03) (Fig. 3). During the consultation of complicated ARI, refusal to dispense drugs to a child that was not physically present increased (3% at baseline vs. 15% post-intervention; *p* = 0.003).

#### Adults with uncomplicated ARI

After the intervention which recommended against dispensing antibiotics, antibiotic dispensing practice remained similar (48% [96/200 consultations] at baseline vs. 40% [75/188 consultations] post-intervention; *p* = 0.1) (Fig. 3).

#### Adults with complicated ARI

After the intervention which recommended referring adult patients with complicated ARI to a health facility for evaluation, antibiotic dispensing increased (44% [44/100 consultations] at baseline vs. 78% [73/94 consultations] post-intervention; *p* = < 0.001) (Fig. 3) and referrals decreased (38% baseline vs. 24% post-intervention; *p* = 0.01) (Fig. 3).

### Post-intervention changes in symptomatic care drugs dispensing

#### Children with uncomplicated ARI

**Fig. 4:**
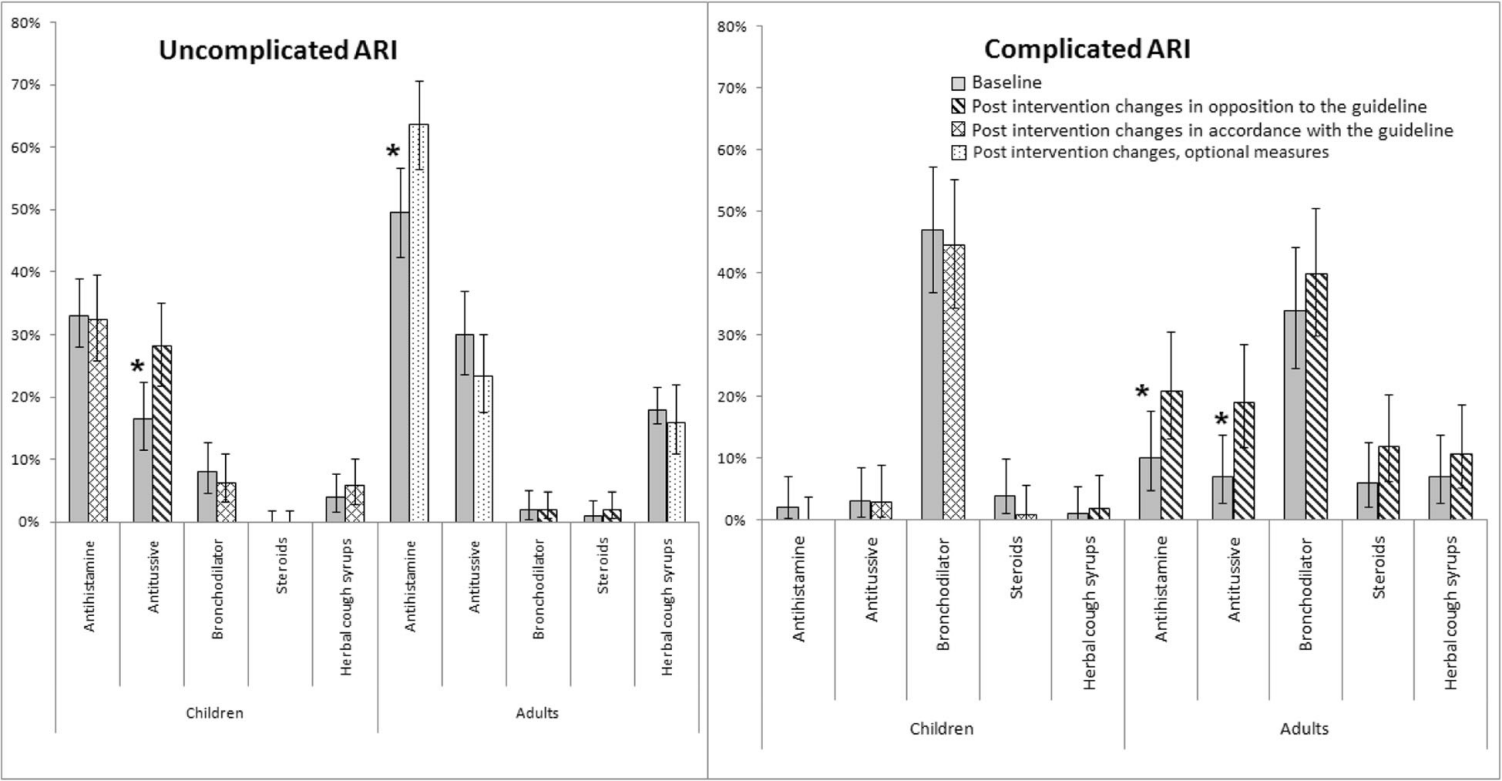
Symptomatic care drugs dispensed for acute respiratory illness (ARI) at baseline and post-intervention in selected Dhaka City pharmacies, June 2012 to December 2013

Although the guidelines recommended against the sale of antihistamines without a physician’s prescription to children aged < 5 years, drug sellers continues to dispense similar amounts of antihistamines (33% at baseline vs. 32% post-intervention; *p* = 0.9). Drug sellers sold increased amounts of antitussives (17% at baseline vs. 28% post intervention; *p* = 0.01), similar amounts of bronchodilators (8% at baseline vs. 5% post-intervention; *p* = 0.2) and herbal cough syrups (4% at baseline vs. 4% post-intervention; *p* = 0.4) for uncomplicated ARI (Fig. 4).

#### Children with complicated ARI

The guidelines discouraged the sale of antihistamines without a physician’s prescription and none of the drug sellers sold these medications to children with complicated ARI after the intervention (2% at baseline vs. 0% post-intervention; *p* = 0.2). The guidelines also discouraged the sale of steroids, but participants rarely sold steroids before and after the intervention (3% at baseline vs. 1% post-intervention; *p* = 0.3). Drug sellers continued to dispense similar amounts of antitussives (3% at baseline vs. 3% post-intervention; *p* = 0.9), bronchodilators (47% at baseline vs. 45% post-intervention; *p* = 0.7) and herbal cough syrups (1% at baseline vs. 2% post-intervention; *p* = 0.3) for complicated ARI (Fig. 4).

#### Adults with uncomplicated ARI

Over the counter drugs (antihistamines, cough expectorants, cough suppressants, acetaminophen, norsol drop) could be used for adults with uncomplicated ARI (Table 2) according to our recommended guidelines. Indeed, antihistamine dispensing increased post-intervention (48% at baseline vs. 64% post-intervention; (*p* = < 0.001). Although the guidelines recommended against prescribing steroids for uncomplicated ARI, drug sellers rarely sold steroids before and after the intervention (1% at baseline vs. 2% post-intervention; *p* = 0.6). Drug sellers also continued to dispense similar amounts of antitussives (30% at baseline vs. 24% post-intervention; *p* = 0.2), bronchodilators (2% baseline vs. 2% post-intervention; *p* = 0.8), and herbal cough syrups (18% baseline vs. 16% post-intervention; *p* = 0.7) for uncomplicated ARI (Fig. 4).

#### Adults with complicated ARI

Although the guidelines only recommended referring adults with complicated ARI to a health facility for evaluation by a physician, drug sellers dispensed more antihistamines (10% at baseline vs. 22% post-intervention; *p* = 0.02) and antitussives (7% at baseline vs. 18% post-intervention; *p* = 0.01) for such patients. Drug sellers also dispensed similar amounts of bronchodilators (34% at baseline vs. 41% post-intervention; *p* = 0.3), steroids (6% at baseline vs. 12% post-intervention; *p* = 0.2) and herbal cough syrups (7% at baseline vs. 11% post-intervention; *p* = 0.4) after the intervention (Fig. 4).

### Advice provided by drug sellers at post-intervention

**Fig. 5:**
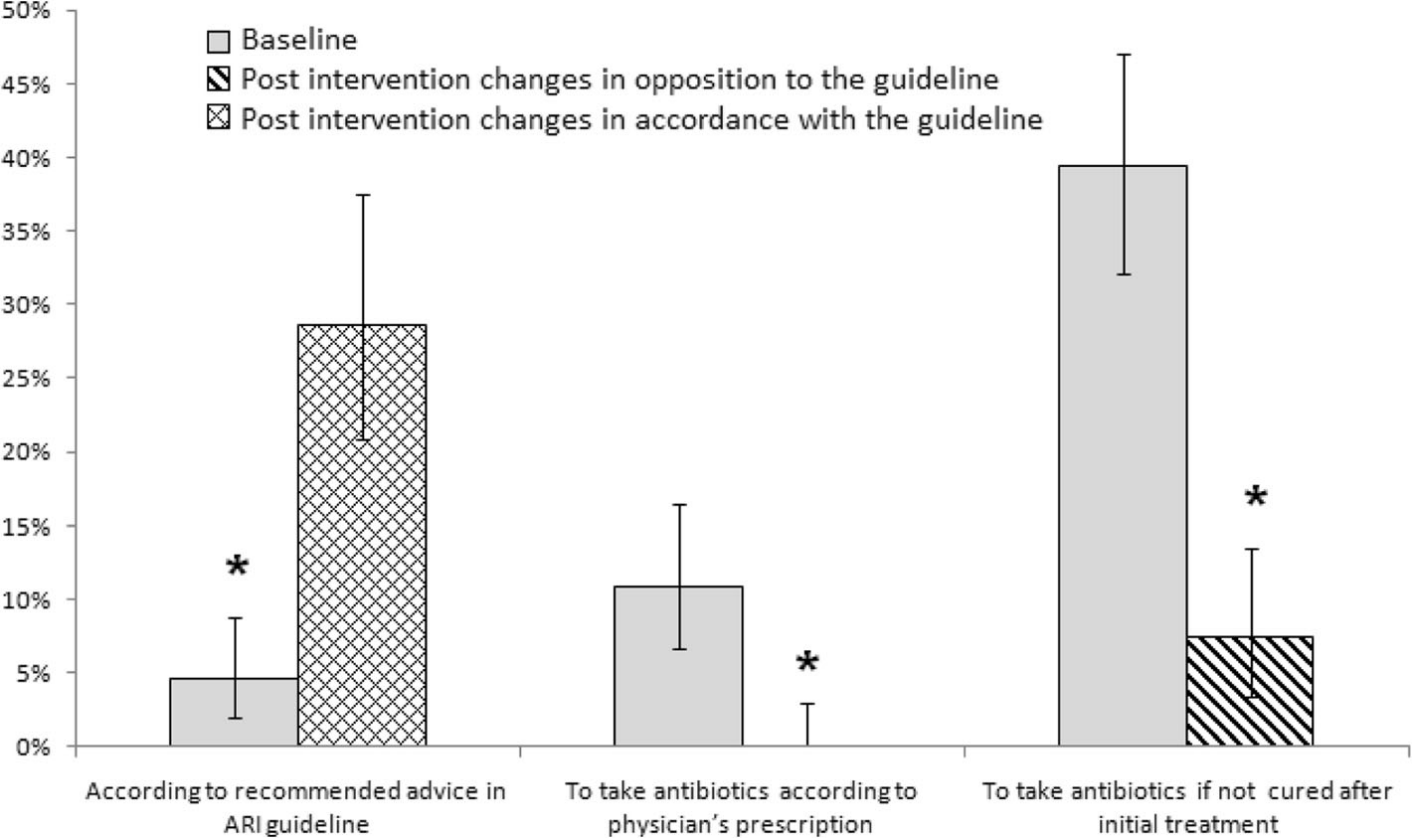
Drug sellers’ advice on acute respiratory illness (ARI) at baseline and post intervention in selected pharmacies within Dhaka City, Bangladesh, June 2012 to December 2013

After the intervention, drug sellers were more likely to provide advice recommended in the guidelines (e.g. keeping babies warm, using acetaminophen for fever, sponging the body for fever, hydrating patients) (5% [8/175 consultations with advice provided] at baseline vs. 29% [35/122 consultations with advice provided] post-intervention; *p* < 0.001). At post intervention, drug sellers were less likely to advice using antibiotics in accordance with a physician’s prescription (11% at baseline vs.0% post-intervention; *p* < 0.001) and advice to take antibiotics if not cured after initial symptomatic treatment at pharmacies (39% at baseline vs. 7% post-intervention; *p* < 0.001) (Fig. 5).Though it was not recommended by the guideline, advice to give nebulized bronchodilators to children with respiratory distress increased post-intervention (7% at baseline vs. 25% post-intervention; *p* < 0.001).

### Post-intervention survey of knowledge, attitudes and practices

**Fig. 6:**
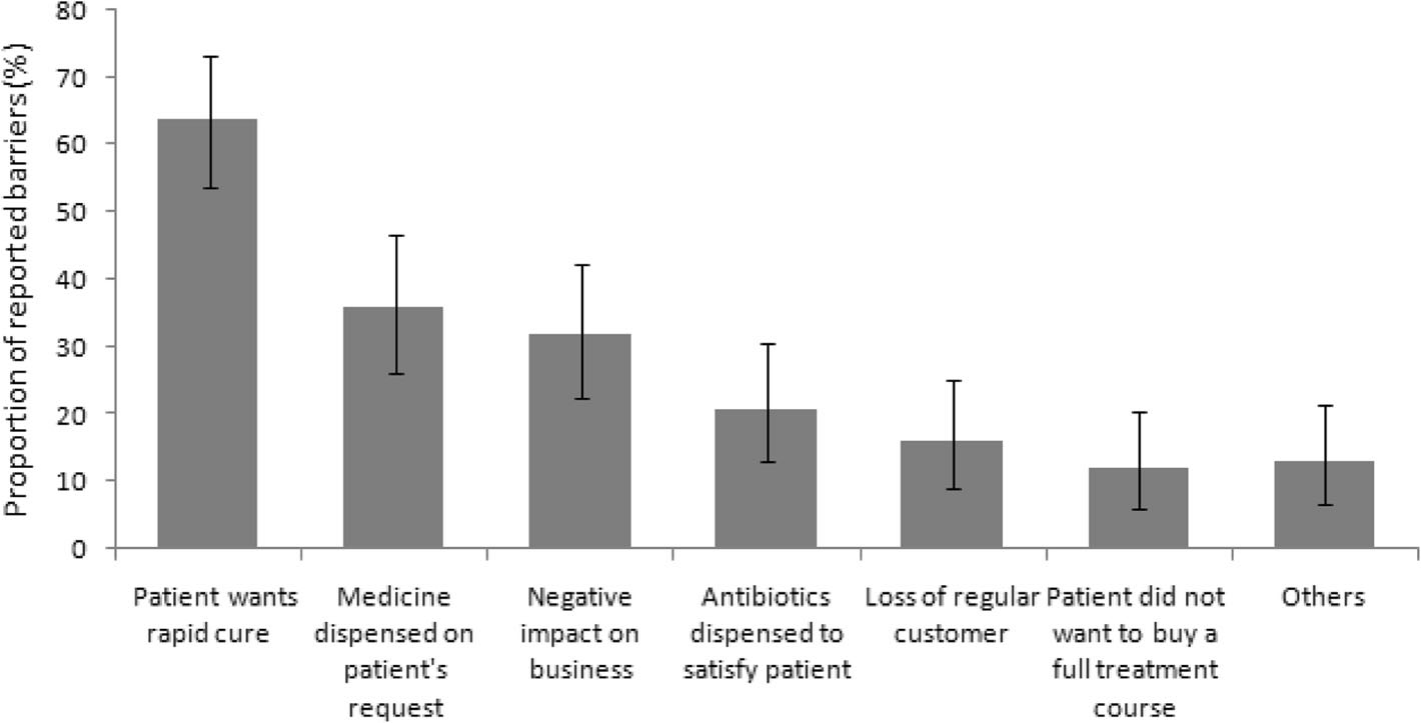
Reasons reported by the drug sellers for not following the guideline (Additional file 3)

Ninety-five drug sellers from 95 pharmacies were interviewed 6 months after the training (two drug sellers were not interviewed because they had quit the pharmacy practice). After the training, ninety-four (99%) stated the training had been useful and 80 (84%) had reviewed the training manual. Posters were observed hanging in 59 (62%) pharmacies. Nevertheless, 87% (83/95) of drug sellers concluded they could not perform all the recommendations in the guideline. Drug sellers cited perceived patients’ desire for rapid cures (64%), patients’ expectations that drug sellers would sell medicine (36%), negative impact on business (32%), and patients’ expectations of receiving antibiotics (21%) among the top reasons for not following the guidelines (Fig. 6).

## Discussion

We evaluated the acceptability of guidelines for the management of ARI and the impact of one-day trainings on drug prescribing and referral patterns of drug sellers in Dhaka, Bangladesh. icddr,b had developed the guidelines for this study purpose in collaboration with partners from the Government of Bangladesh, IEDCR. After receiving a one-day training on the ARI guidelines, fewer drug sellers dispensed antibiotics for uncomplicated ARI in accordance with the guidelines, but there was no improvement in the management of children with complicated ARI. According to the guidelines, drug sellers should have only dispensed antibiotics after assessing children for breathing difficulty because this could signal pneumonia. In addition, if the children were not present for the assessment, drug sellers should recommend caregivers bring their children for clinical assessment and to provide appropriate management or referral. In our study, approximately 1/6 of drug sellers seemed reluctant to dispense drugs to absent children because they could not assess their clinical condition.

While the guidelines attempted to discourage antibiotic use among adults without a prescription, antibiotic dispensing remained similar for adults with uncomplicated ARI and increased significantly for complicated ARI. According to the guidelines, drug sellers should have referred adults with complicated ARI to a health facility for evaluation and treatment by a physician. Instead, our study found that drug sellers referred fewer patients to physicians at post-intervention. Drug sellers seemed reluctant to refer patients to physicians for fear of loss of business. A possible explanation for this behavior might be that referred physicians may not recommend antibiotics, and if they do, the patient may not return to the same pharmacy to buy medicine. To avoid a negative impact on their business and their relationship with their customer base, it appeared drug sellers were reluctant to suggest referral as they were concerned that patients may go to another pharmacy to seek further health care.

Drug sellers dispensed drugs that could relieve patients’ symptoms. In addition, drug sellers believed that refusal to dispense drugs would have a negative impact on their relationship with customers and decrease profits. Thus, it is not surprising that drug sellers dispensed antihistamines more often for adults with uncomplicated and complicated ARI, and antitussives to adults with complicated ARI. In contrast, the guidelines did not recommend antihistamines and antitussive drugs for children aged less than five years [39, 40], but our recommendation to reduce dispensing did not change dispensing practices among children.

Drug sellers cited perceptions of customer dissatisfaction in addition to potential negative impact on their business as the key barriers to following the guidelines. Drug sellers asserted that customers expected antibiotics for rapid cure. Although none of our staff requested antibiotics, drug sellers seemed to assume that customers wanted antibiotics. These beliefs are shared among drug sellers in other cultures [41]. Studies in Qatar and India found that drug sellers cited perceived patient demand and the desire to make a profit as the reasons for inappropriate dispensing practices irrespective of drug sellers’ knowledge about the recommended management of common ailments, including ARI [42, 43]. Although several studies have reported a positive impact of educational interventions on drug sellers’ management of common ailments [33, 44–46], our educational intervention only seemed to prevent antibiotic dispensing among children with uncomplicated ARI. Our findings are similar to a study in Uganda where face-to-face educational interventions did not improve ARI management [35]. Our intervention might have made drug sellers more aware of the potential dangers of ARI, but was insufficiently compelling to change their business model. Although > 80% of ARIs are caused by viruses, 85% of Bangladeshi patients with ARI reported receiving antibiotics from physicians [47]. One educational session might be insufficient to change drug sellers’ behavior, particularly when loss of profits is a concern. For the guidelines to be adopted, drug sellers may have to first develop a long-term relationship with physicians so that physicians routinely send patients to them with a prescription for medication and create a positive feedback loop for appropriate referrals.

Furthermore, lack of awareness among the customers about the appropriate use of antibiotics might be an important driver of inappropriate dispensing. Nevertheless the public often believes that an ARI should be treated with antibiotics to rapidly cure the illness [48, 49]. Misuse of antibiotics is costly, may lead to adverse events [50], and antimicrobial resistance [51]. Raising public awareness about the appropriate use of antibiotics and the risks of antimicrobial resistance is necessary [52].

The dispensing practices we documented are typical of those previously observed in Bangladesh and in other low and middle-income countries [53]. It is also probable that a single day intervention training alone was insufficient to see any potential lasting effects to improve ARI management. Moreover, the guidelines and teaching materials could have been clearer and better organized as more effective tools for behavior change. A longer training and at regular intervals with an enforced annual licensing and reaccreditation system might provide a greater incentive to change dispensing practices. Additionally, regular comprehensive monitoring and supervision with adequate resource personnel is needed to identify unlicensed and non-compliant pharmacies.

### Limitations

We evaluated the effectiveness of the intervention through a cross-sectional study rather than through a randomized double blinded intervention trial which might have reduced bias. Moreover, we deployed surrogate caregivers to study drug seller dispensing practices. These field staff might not have represented the subtleties of real-life and were not accompanied by a sick individual which might have influenced the pharmacist to act differently.

## Conclusion

Except for reducing non-recommended antibiotic use among children with uncomplicated ARI, our training sessions, laminated posters or cards, training manuals and guidelines did not improve ARI case-patient management. It is possible that improving the teaching materials and sessions would lead to a more effective intervention. Our findings also suggest that customer demand may also drive antibiotic dispensing among the drug sellers. Public health authorities should explore the potential value of pairing educational interventions among drug sellers with raising awareness about proper antibiotic use among general population. In addition, it might be helpful to improve the ARI management in pharmacies through the enforcement of annual licensing and reaccreditation system. Systematic comprehensive monitoring by DGDA followed by penalties for non-compliant pharmacies could improve drug seller practices Additional studies are needed to explore whether making re-licensing contingent on the completion of annual pharmacy education credits would improve dispensing and referral practices.

## Additional files

Additional file 1: Poster/Flip chart “Key messages through educational intervention on acute respiratory illness (ARI) management for the drug sellers at pharmacy”. “We had provided a poster to hang in the pharmacy and a flip chart to keep in the pharmacy desk to each drug sellers of the selected study pharmacies with the key messages from the training/education intervention on ARI management” (PDF 72 kb)

Additional file 2: Training Manual “Awareness guideline on Acute Respiratory Illness for the drug sellers”. “We have provided this training manual to each drug sellers of the selected study pharmacies which contained details material of the training/education intervention on ARI management” (PDF 69 kb)

Additional file 3: Questionnaire for drug sellers after educational intervention. “This was the questionnaire for post intervention survey on knowledge, attitude and practice of the drug sellers to collect information on drug sellers compliance with the educational intervention” (PDF 117 kb)

## Abbreviations

ARI: Acute Respiratory Illness
CDC: Centers for Disease Control and Prevention
DGDA: Directorate General of Drug Administration
icddr,b: International Centre for Diarrhoeal Disease Research, Bangladesh
IEDCR: Director of the Institute of Epidemiology, Disease Control & Research
IMCI: Integrated Management of Childhood Illness
IRB: Institutional Review Boards
LMAF: Local Medical Assistant and Family Planning
WHO: World Health Organization
