# Supplementary material for: Effectiveness of an educational intervention to improve antibiotic dispensing practices for acute respiratory illness among drug sellers in pharmacies, a pilot study in Bangladesh
Source: BMC Health Serv Res. 2018 Aug 31;18:676. doi: 10.1186/s12913-018-3486-y (PMC6119333; doi:10.1186/s12913-018-3486-y)
Supplement: Supplementary file 1 — Poster/Flip chart “Key messages through educational intervention on acute respiratory illness (ARI) management for the drug sellers at pharmacy”. “We had provided a poster to hang in the pharmacy and a flip chart to keep in the pharmacy desk to each drug sellers of the selected study pharmacies with the key messages from the training/education intervention on ARI management” (PDF 72 kb) [file 12913_2018_3486_MOESM1_ESM.pdf]

Appendix 1: Poster/Flip chart "Key messages through educational intervention on acute respiratory illness (ARI) management for the drug sellers at pharmacy"

Symptoms of acute respiratory illness (for both children and adults):

- Runny nose, stuffy nose or nasal congestion, cough, breathing difficulty

Any of these above symptoms with or without the symptoms below

- Fever, sore throat, sneezing

Advice about acute respiratory illness:
<u>What to do</u>
For children (2 months to 5 years):

- **Keep the baby warm in winter**

For children (>2 months) and adults:

- **If febrile, give acetaminophen, sponge the whole body, and encourage drinking of plenty of water for patients 6 months and above.**
- **Also, follow-up in 2 days if complicated ARI or 5 days if uncomplicated ARI and symptoms have not improved**

Advice about acute respiratory illness:
<u>What should NOT be done/given</u>

For children (2 months to 5yrs):

- Antibiotic except for pneumonia,
- Medicines containing Pseudoephedrine
- Antihistamine
- Changing regular food or stop breast feeding in breastfed baby

For children (2 months and older) and adults:

- Steroid, NSAID (non steroidal anti-inflammatory drug)/Pain killer for fever

For all children aged <2 months, refer to physician.
For children aged 2 months to <5 years, see box below for referral guidelines.
For children (2 months and older) and adults:

- Severe fever, chest pains, or headaches
- **Breathing difficulties** such as wheezing or shortness of breath (for children >5yrs of age and adults)
- Blood with cough,
- Cough >**3 consecutive weeks**

Contact physician

Cough and or runny nose and fever (adults): Over the counter drugs as cough syrups and antihistamines can be given with acetaminophen if fever.
Advice: To relieve the cough **drink warm lime water, honey, lemon tea or warm salt water gurgling**

**Classification of cough or breathing difficulty among children <5 years**		
**Severe pneumonia Or Very severe disease**<br><br>**Any general danger sign or chest indrawing**<br><br>***General Danger signs:**<br>▪ Inability to drink or breastfeed, Repeated vomiting, Convulsion, Lethargy/abnormally sleepy/unconscious<br><br>**Management**<br>Refer **URGENTLY** to physician/hospital	**Pneumonia**<br><br>**Fast breathing**<br>▪ 2 months to 12 months- 50 or more respiration per minute<br>▪ 12 months to 59 months-40 or more respiration per minute<br>▪ **Management** Antibiotic (Oral **Amoxycillin**) for 5 days<br>▪ Advise the caretaker to return immediately if the child develops danger signs<br>▪ Follow-up in **2 days**	**No pneumonia: cough or cold**<br><br>No signs of pneumonia or very severe disease<br>▪ **Management** If coughing for more than **3 weeks** contact physician<br>▪ To relieve the cough **drink warm lime water, honey, lemon tea**<br>▪ Advise the mother when **(*danger sign**) to return immediately<br>▪ Follow up in **5 days** if not improving

Over the counter drugs: For acute respiratory illness (For adults)

- Dextromethorphan (Cough suppressants)
- Guaiphenesin+pseudoephedrine (Cough expectorants)
- Loratadine (Non-sedating antihistamine)
- Chloropheniramine (Sedating antihistamine)
- Diphenhydramine (Sedating antihistamine)
- Promethazine (Sedating antihistamine)
- Paracetamol
- Norsol drop
