# Supplementary material for: Effectiveness of an educational intervention to improve antibiotic dispensing practices for acute respiratory illness among drug sellers in pharmacies, a pilot study in Bangladesh
Source: BMC Health Serv Res. 2018 Aug 31;18:676. doi: 10.1186/s12913-018-3486-y (PMC6119333; doi:10.1186/s12913-018-3486-y)
Supplement: Supplementary file 2 — Training Manual “Awareness guideline on Acute Respiratory Illness for the drug sellers”. “We have provided this training manual to each drug sellers of the selected study pharmacies which contained details material of the training/education intervention on ARI management” (PDF 69 kb) [file 12913_2018_3486_MOESM2_ESM.pdf]

Appendix 2: Training Manual "Awareness guideline on Acute Respiratory Illness for the drug sellers"

Acute respiratory infection is an acute infection of the upper or lower respiratory tract causing common cold to pneumonia.

Most cases of ARI are due to viral infections. But some cases are due to bacterial infection, while others may be complicated by bacterial super infections in which a bacterial infection develops after a viral infection.

- **Symptoms of acute respiratory infection**
    - Runny nose
    - Stuffy nose or nasal congestion or nose block
    - Cough
    - Breathing difficulty

A person will be diagnosed as a case of acute respiratory illness if he/she comes with any of the above respiratory symptoms with or without fever or any other listed symptoms below.

- Sore throat
- Sneezing
- Watery eye/redness of the eye
- Bodyache
- Headache

NB: ARI may cause death in children under- five years of age if not properly diagnosed and treated.

- **Proper history taking (Explaining the importance of each step of history taking)**

Before starting to take history ask for the age of the patient.

- **Duration of cough**
    - Usually cough may last up to 1-2weeks. But in some cases it may last up to 4 weeks after the infection has gone.
    - Cough more than 3 weeks needs tuberculosis (TB) exclusion.

- **Nature of cough**

- A person having cough with breathing difficulty such as wheezing or shortness of breath, or cough mixed with blood must contact with physician.

- **Duration of fever**

- Usually fever may persist for 3 to 7 days. Fever more than 7 days needs consultation with a physician to evaluate the cause of fever.

- **Nature of fever**

- Advise to record the temperature.

- **Any other related illness**

- Advise to consult a physician if acute respiratory infection is associated with diarrhoea or any severe disease.

- **Any other associated illness**

- Ask for any other associated illness like gastric ulcer, asthma, diabetes, high blood pressure or heart disease. Advise to contact with a physician for the associated illness.

- **Specific known allergy to any medication**

- Sometimes patient may have history of allergic reaction to certain medication like penicillin, cotrimoxazole. So drug seller should be aware of drug allergy before dispensing medicines.

- **Ask for the following danger signs for under-five children**

- Inability to drink or breastfeed
- Convulsion
- Lethargy/abnormally sleepy/unconscious
- Repeated vomiting

NB: Immediately refer the child to nearby hospital after giving first dose of antibiotic.

- **Clinical Examination**

  - **Temperature**
    - If possible temperature should be measured if a patient complains of fever. Axillary temperature 100·4 °F or more should be assessed as fever.

If a mother or caregiver of a child complains of respiratory distress of the child, the drug seller should count the respiratory rate and look for chest in-drawing and other danger signs.

- **Respiratory rate count**
  - Respiratory rate should be counted with a watch having a second hand or a digital watch for 1 minute (60 seconds). The child should be calm and quiet before counting the respiratory rate. Respiratory rates below these age-specific cut-offs will be counted as normal. Any child having a rapid respiratory rate would be diagnosed as pneumonia.

**If child aged:**	**Child has rapid respiration if:**
Birth to 2 months	60 or more per minute
2 months to 12 months	50 or more per minute
12 months to 5 years	40 or more per minute

- **Chest in-drawing**
  - Chest in-drawing is defined as a definite inward movement of lower chest wall while breathing in (inspiration). For observing chest in-drawing, the child should be made to lie flat in bed or in the mother's lap. Whenever there is chest in-drawing (with or without fast breathing), severe pneumonia should be diagnosed. The in-drawing must be present with every breath.

- **Look and ask for danger signs** (inability to drink, repeated vomiting, convulsion, lethargy/abnormally sleepy/unconscious)

- **Diagnosis (Children under-five years)**

  - Very severe disease or Severe Pneumonia
    - If the patient has chest in-drawing with or without fast breathing with any of the danger signs will be diagnosed as a case of severe pneumonia or very severe disease.
  - Pneumonia
    - Respiratory rate above the age specific cut of value will be diagnosed as a case of pneumonia.
  - No pneumonia (Cough and cold)
    - Respiratory rate below the age specific cut-off value will be diagnosed as a case of "No pneumonia", only cough and cold.

- **Patient Management**

  - If the patient has fever give paracetamol (Proper advice for dose and duration of paracetamol). Paracetamol can also be used in case of myalgia/bodyache/headache.

    Paracetamol dose in adult: 1-2 tablet every 6 hours as required
    Paracetamol dose in children: Up to 1 year of age (0.5 to 1 teaspoon every 6 hours)

    1 year to 5 years of age (1 to 2 tea spoons every 6 hours as required)

- **If the child is diagnosed as a case of pneumonia**
  - Oral antibiotic (**Amoxycillin**) for 5 days
  - To relieve the cough advise to **drink warm lime water, honey, lemon tea or warm salt water gurgling**
  - Advise the mother when (i.e., if there are **danger signs**)to return immediately
  - Follow-up in **2 days**

- **If the child is diagnosed as No pneumonia (cough and cold)**
  - If coughing for more than **3 weeks** refer for to hospital for diagnosis
  - To relieve the cough advise to **drink warm lime water, honey, lemon tea or warm salt water gurgling**
  - Advise the mother when (i.e., if there are **danger signs**)to return immediately
  - Follow up in **5 days** if not improving

- **Advice/what to do**
  - Advise to keep the baby **warm**
  - Advise the patient to drink plenty of **water**
  - Advise to **sponge whole body** with warm water if high fever (101 °F or higher)
  - Advise to follow up in **5 days** if not improving
  - Advise on **danger sign**
  - To relieve the cough Advise to **drink warm lime water, honey, lemon tea or warm salt water gurgling**

- **What should be done:**
  - A**ntibiotic** should not be used except pneumonia in children
  - Don't use **steroid**
  - Don't use **pseudoephedrine** containing drugs for children
  - Don't use **antihistamine** for children
  - Don't use non-steroidal anti-inflammatory drugs (**NSAID**; e.g., ibuprofen) for fever
  - Advise not to use **warm clothes** or covering if has a fever
  - Advise not to change **breast feeding or food**
  - Advise to **stop smoking** if cough is present

- **Proper referral**
  - Refer urgently to Thana health complex/ hospital /physician if severe pneumonia or very severe disease in children or adult patient having respiratory distress.

Classification table for cough or breathing difficulty among under-five children

SIGNS	CLASSIFY AS	TREATMNT
• Any general danger sign or chest indrawing	Severe Pneumonia<br>Or<br>Very severe disease	• Ensure appropriate diet to prevent **hypoglycaemia**<br>• Refer **URGENTLY** to physician/hospital<br>• If possible contact physician before referring
• Fast breathing	Pneumonia	▪ Oral antibiotic (Amoxycillin) for 5 days<br>▪ To relieve the cough advise to **drink warm lime water, honey, lemon tea or warm salt water gurgling**<br>▪ Advise the mother when (if a **danger sign** is present)to return immediately<br>▪ Follow-up in **2 days**
▪ No signs of pneumonia or very severe disease	No Pneumonia: Cough or Cold	• If coughing for more than **3 weeks** refer for to hospital for diagnosis<br>▪ To relieve the cough advise to **drink warm lime water, honey, lemon tea or warm salt water gurgling**<br>▪ Advise the mother when (**danger sign**)to return immediately<br>• Follow up in **5 days** if not improving

- **Contact physician –**
  - If the patient is a child under 2 months of age
  - If symptoms such as fever, chest pains, or headaches become **worse or severe**.
  - If develop **breathing difficulties** such as wheezing or shortness of breath.
  - If **cough up blood**. Blood may be bright red but dark or rusty colored sputum may indicate blood.
  - If become **drowsy or confused**.
  - If cough that persists for **longer than 3 weeks**.

Appropriateness of the dispensed medicine
World Health Organization defined rational use of drugs as follows:
"Patients receive medications appropriate to their clinical needs, in doses that meet their own individual requirements, for an adequate period of time, and at the lowest cost to them and their community".

The overuse, underuse or misuse of medicines results in wastage of limited resources and health hazards. Examples of irrational use of medicines include:

- Use of too many medicines per patient "poly-pharmacy"
- Inappropriate use of antibiotics
- Inadequate dosage
- Antibiotic use for non-bacterial infections
- Over-use of injections when oral formulations would be more appropriate
- Failure to prescribe in accordance with clinical guidelines
- Inappropriate self-medication

Antibiotic use:
Irrational use of antibiotics leads to antimicrobial resistance (AMR). Irrational use of antibiotics means misuse of antibiotics in case of non-bacterial infection (such as viral respiratory illness), inappropriate dose and duration. Antimicrobial resistance is resistance of a microorganism to an antimicrobial medicine to which it was previously sensitive. Due to AMR, standard treatment becomes ineffective and so infection persists and may spread to others.
So, in conclusion: Inappropriate and irrational use of antimicrobial medicines provides favourable conditions for resistant microorganisms to emerge, spread and persist.

Steroid use:
There is absolutely NO place for any sort of corticosteroids (dexamethasone, betamethasone, and prednisolone) use in the treatment of fevers or respiratory distress due to acute respiratory illness. They compromise the body immune system even further, thereby increasing the risk of

infections (bacterial super infection) that may be almost impossible to cure even with the latest generation of antibiotics. Patient may worsen, even there might be some symptomatic improvement.

Medicine used to treat allergy (Antihistamine):
Antihistamine should not be used among children under five years, as it may increase chest congestion and may lead to asthma.
Antihistamines may be used in adult with allergic rhinitis but not viral rhinitis. Allergic rhinitis can be diagnosed as: runny nose for about a month or rhinitis in any specific allergic condition.

Cough expectorants and cough suppressants:
Evidence from different studies showed that the use of cough expectorants or cough suppressants has no beneficial effect on the common cold or acute cough in adult or children.

Hygiene practice for prevention of disease transmission:
Hand washing should be practiced regularly with soap water to prevent acute transmission of infection.

Over The Counter drugs: For Acute Respiratory Illness (For adults)

- Dextromethorphan (Cough suppressants)
- Guaiphenesin+pseudoephedrine (Cough expectorants)
- Loratadine (Non-sedating antihistamine)
- Chloropheniramine (Sedating antihistamine)
- Diphenhydramine (Sedating antihistamine)
- Promethazine (Sedating antihistamine)
- Paracetamol
- Norsol drop
