# Supplementary material for: Effectiveness of an educational intervention to improve antibiotic dispensing practices for acute respiratory illness among drug sellers in pharmacies, a pilot study in Bangladesh
Source: BMC Health Serv Res. 2018 Aug 31;18:676. doi: 10.1186/s12913-018-3486-y (PMC6119333; doi:10.1186/s12913-018-3486-y)
Supplement: Supplementary file 3 — Questionnaire for drug sellers after educational intervention. “This was the questionnaire for post intervention survey on knowledge, attitude and practice of the drug sellers to collect information on drug sellers compliance with the educational intervention” (PDF 117 kb) [file 12913_2018_3486_MOESM3_ESM.pdf]

Appendix 3: Questionnaire for drug sellers after educational intervention

Dispensing practices and impact of educational intervention for acute respiratory illness among drug sellers, A pilot study in Bangladesh

বাংলাদেশে ঔষধ বিক্রেতাদের মধ্যে শ্বাসতন্ত্রের প্রদাহ জনিত রোগে ঔষধ-বিতরণ প্র্যাকটিস এবং তাদের মধ্যে এ রোগের চিকিৎসা সংক্রান্ত শিক্ষামূলক মধ্যবর্তিতার প্রভাব: একটি প্রাথমিক সমীক্ষা

ক্রমিক নং	প্রশ্ন	উত্তর	নির্দেশনা
**101.**	ফার্মেসীর নাম এবং কোড নম্বর<br>(Name of the Pharmacy & code):		
**102.**	প্রশিক্ষন প্রাপ্ত ঔষধ বিক্রেতার নাম:<br>(Trained drug seller's Name):		
**103.**	সাক্ষাৎকার গ্রহণকারীর নাম এবং কোড নম্বর:<br>(Interviewers name & code)		
**104.**	ভিজিটের তারিখ (Date of visit):		
**105.**	সুপারভাইজারের নাম, স্বাক্ষর<br>(Name of supervisor & Signature)<br>তারিখ: ----/----/2013		
**106.**	ডাটা এন্ট্রিকারীর নাম, স্বাক্ষর<br>(Data Entry Person & Signature)<br><br>তারিখঃ ----/----/2013		
**201.**	আপনি কি শ্বাসতন্ত্রের প্রদাহ জনিত রোগের ব্যবস্থাপনা বিষয়ক প্রশিক্ষণে অংশগ্রহন করেছেন ?<br>(Did you attend the training on Management of acute respiratory Illness?)	Yes (হ্যাঁ)= 1<br>No (না) = 2<br>Not applicable (জানি না) = 3	
**202.**	আপনি কি মনে করেন প্রশিক্ষণ টি উপকারী ছিল?<br>(Do you think it was useful?)	Yes (হ্যাঁ)= 1<br>No (না) = 2<br>Not applicable (জানি না) = 3	যদি উত্তর "না" হয়, তহলে **"204"** নম্বর প্রশ্ন করুন এবং যদি উত্তর "হ্যাঁ" হয়,তাহলে **"203"** নম্বর প্রশ্ন করুন<br>If no skip question **"203"** and if yes skip **"204"**

**203.**	যদি হ্যাঁ হয়, কেন (বর্ণনা করুন)? (If yes, why) **Please Probe this Question:**<br>--		
**204.**	যদি না হয়, কেন (বর্ণনা করুন)? (If no, why) **Please Proving this Question:**<br>--		
**205.**	আপনাকে ট্রেনিং-এ যে নির্দেশিকা টি দেয়া হয়েছিল সেটি কোথায় সংরক্ষণ করেন / রেখেছেন ?(where do you keep the manual which was Provided during training?	বাসায় = 1<br>দোকানে = 2<br>চেম্বারে = 3<br>বন্ধুর কাছে = 4<br>হারানো গিয়াছে = 5<br>☐	
**206.**	নির্দেশিকা টি পড়ে দেখেছিলেন কি ?<br>(Did you read the manual?)	Yes (হ্যাঁ) = 1<br>No (না) = 2<br>Not applicable (জানি না) = 3<br>☐	উত্তর না হলে **"207"** নং প্রশ্ন করার প্রয়োজন নাই।
**207.**	নির্দেশিকা টি পড়তে বা বুঝতে কোন সমস্যা আছে ?<br>(Do you feel any problem to read and understand the manual)	Yes (হ্যাঁ)= 1<br>No (না) = 2<br>Not applicable (জানি না) = 3<br>☐	উত্তর **হ্যাঁ** হলে **"208 "** নং প্রশ্ন করুন।
**208.**	নির্দেশিকা টিতে কি সমস্যা আছে বলে আপনার মনে হয় ? **Please Probe this Question:**<br>**(What problems are there in the manual)**<br>--		
**209.**	আপনি কি নির্দেশিকা টি সম্পূর্ণ মেনে চলেন?<br>(Do you follow the manual completely)	Yes (হ্যাঁ)= 1<br>No (না) = 2<br>Not applicable (জানি না) = 3<br>☐	
**210.**	যদি না হয়, কেন (বর্ণনা করুন)? (If not, why) **Please Proving this Question:**<br>--		
**211.**	আপনি কি মনে করেন নির্দেশিকাটি সম্পূর্ণ মেনে চলতে কোন বাঁধা আছে?<br>(Do you think there are any barriers that prevent you from following the manual)	Yes (হ্যাঁ)= 1<br>No (না) = 2<br>Not applicable (জানি না) = 3<br>☐	
**212.**	যদি হ্যাঁ হয়, সেটা কি ? (If yes, what is it) **Please Proving this Question:**<br>--		
**213.**	পোষ্টার টি ঝুলন্ত অবস্থায় আছে কি না? **(পর্যবেক্ষণ)**<br>(Is the poster hanging in the pharmacy) (Observe)	Yes (হ্যাঁ)= 1<br>No (না) = 2<br>☐	

"আপনার মূল্যবান সময় আমাদের দেয়ার জন্য আপনাকে অনেক ধন্যবাদ"

"Thank you for providing us your valuable time"
